# Low zinc status and long-term risk of incident sepsis in patients with type 2 diabetes: a multicenter cohort study

**DOI:** 10.3389/fnut.2026.1897312

**Published:** 2026-08-21

**Authors:** Kuo-Chuan Hung, Ting-Sian Yu, Yi-Chen Lai, Wei-Cheng Liu, I-Wen Chen

**Affiliations:** 1 Department of Anesthesiology, Chi Mei Medical Center, Tainan City, Taiwan; 2 School of Medicine, College of Medicine, National Sun Yat-sen University, Kaohsiung City, Taiwan; 3 Department of Anesthesiology, E-Da Hospital, I-Shou University, Kaohsiung City, Taiwan; 4 Department of Anesthesiology, Chi Mei Hospital, Liouying, Tainan City, Taiwan

**Keywords:** infection, propensity score matching, sepsis, TriNetX, type 2 diabetes mellitus, zinc deficiency

## Abstract

**Background:**

Zinc deficiency is common in patients with type 2 diabetes mellitus (T2DM) and is associated with an increased susceptibility to infection. However, because sepsis reflects a dysregulated host response to infection, whether low zinc status is more strongly associated with sepsis than with nonsepsis infections in patients with T2DM remains unclear.

**Methods:**

This retrospective cohort study used the TriNetX Global Collaborative Network to identify adult patients with T2DM and documented zinc measurements between 2010 and 2024. Patients were classified into low zinc status (<0.70 μg/mL) and normal zinc status (0.70–1.20 μg/mL) groups. The primary outcome was incident overall sepsis at five-year follow-up, comprising severe and other types of sepsis. Secondary outcomes included severe sepsis, bacteremia, all-cause mortality, and non-sepsis infections, defined as a composite of pneumonia, urinary tract infection, cellulitis, and abdominal infections. An exposure-gradient analysis was performed to compare severe zinc deficiency (<0.50 μg/mL) with normal zinc status.

**Results:**

After matching, 9,856 pairs were analyzed using propensity score matching. Low zinc status was associated with a higher risk of overall sepsis (HR, 1.37; 95% CI, 1.23–1.54), severe sepsis (HR, 1.30; 95% CI, 1.10–1.55), bacteremia (HR, 1.23; 95% CI, 1.02–1.48), and all-cause mortality (HR, 1.56; 95% CI, 1.41–1.73). The association with non-sepsis infections was smaller (HR, 1.08; 95% CI, 1.03–1.13). The association between low zinc status and overall sepsis remained consistent across sensitivity and subgroup analyses (HR range, 1.31–1.44). In the exposure-gradient analysis, severe zinc deficiency showed a stronger association with overall sepsis than with non-sepsis infection (HR, 1.98 vs. 1.21).

**Conclusion:**

Low zinc status was associated with a higher long-term risk of incident sepsis in patients with T2DM, and the magnitude of association appeared to be greater for sepsis than for non-sepsis infections. These findings are hypothesis-generating and suggest that low zinc status may serve as a marker of sepsis vulnerability in patients with T2DM.

## 1 Introduction

Type 2 diabetes mellitus (T2DM) is a well-established risk factor for sepsis, with patients with diabetes experiencing a higher incidence of sepsis and greater sepsis-related mortality than the general population (1–4). This heightened susceptibility has been attributed to chronic hyperglycemia-induced immune dysfunction, including impaired neutrophil chemotaxis, defective phagocytosis, and a sustained proinflammatory milieu (1, 5–8). Identifying modifiable factors that contribute to sepsis risk in this vulnerable population remains a priority.

Zinc is an essential trace element that serves a dual role in host defense: it supports pathogen clearance through innate and adaptive immune effector functions and, critically, restrains excessive inflammatory activation by modulating the nuclear factor-kappa B signaling cascade and cytokine release (9–12). During systemic inflammation, pro-inflammatory cytokines, particularly interleukin-6, upregulate hepatic ZIP14 and promote the redistribution of circulating zinc to the liver, thereby lowering serum zinc concentrations (9–11). This cytokine-driven redistribution is especially relevant in sepsis, in which cytokine overproduction is a hallmark of the dysregulated host response (13–15). Zinc deficiency is highly prevalent in patients with T2DM (16, 17). Chronic hyperglycemia may impair renal tubular zinc reabsorption, thereby increasing urinary zinc excretion. Insulin resistance and low-grade systemic inflammation may also alter the expression of zinc transporters, including ZIP14, while impaired intestinal absorption may further disrupt zinc homeostasis (16–18). Low zinc status has been consistently linked to an increased susceptibility to common infections, such as pneumonia and urinary tract infections (9, 19–22). However, whether a low zinc status is more strongly associated with sepsis than with non-sepsis infections remains uncertain. Comparing these outcomes may clarify whether low zinc status marks sepsis vulnerability beyond general susceptibility to infection.

This study used a large multicenter federated research network to examine the association between low zinc status and 5-year risk of incident sepsis in patients with T2DM. By evaluating both sepsis and non-sepsis infectious outcomes, we sought to compare the relative magnitude of their associations with a low zinc status. This approach was intended to clarify whether a low zinc status may serve as a marker of greater sepsis vulnerability, rather than merely reflecting a general susceptibility to infection.

## 2 Methods

### 2.1 Data source and ethical statement

This retrospective cohort study used the TriNetX Global Collaborative Network, a federated research platform that aggregates de-identified electronic health record data from over 100 healthcare organizations in multiple countries. The platform enables real-time cohort identification, propensity score matching, and survival analyses, while the data remain within each contributing institution. The TriNetX platform has been widely used in numerous peer-reviewed studies across diverse clinical disciplines (23–26). As the study used only de-identified data, the requirement for informed consent was waived. Ethical approval for the study protocol was obtained from the Institutional Review Board of Chi Mei Medical Center (Tainan, Taiwan). This study adhered to the principles of the Declaration of Helsinki and the applicable institutional standards.

### 2.2 Exposure definition

Adult patients aged ≥18 years with a diagnosis of type 2 diabetes mellitus (T2DM) were retrieved from the TriNetX Global Collaborative Network covering the period from January 1, 2010, to December 31, 2024. No upper age limit was set. Eligible patients were required to have at least one serum or plasma zinc concentration recorded in the structured laboratory data set. Zinc measurements were performed in local laboratories of the participating healthcare organizations as part of routine clinical care. Because TriNetX provides de-identified, harmonized laboratory results, assay-specific information, including the analytical technique, instrument platform, calibration procedure, and local quality-control protocol, was not available.

The index date was defined as the date of qualifying zinc measurements. Patients were assigned to the low zinc cohort if their zinc level was <0.70 μg/mL and to the reference cohort if their zinc level was within the normal range of 0.70–1.20 μg/mL. These thresholds were selected based on commonly used clinical reference ranges for circulating zinc, with concentrations below 0.70 μg/mL (70 μg/dL) indicating low zinc status and concentrations of 0.70–1.20 μg/mL representing the commonly used normal range (27). To guard against exposure misclassification, any patient in the low zinc cohort with a normal zinc measurement in the 3 years before cohort entry was excluded; reference cohort patients with a low zinc measurement over the same look-back period were excluded on the same basis.

### 2.3 Exclusion criteria

To restrict the analysis to incident cases, we excluded patients with a recorded diagnosis of sepsis or severe sepsis in the year preceding the index zinc measurement. This 1-year washout period was selected to reflect a clinically relevant history of recent sepsis while avoiding the excessive exclusion of remote sepsis events that may no longer represent an unstable baseline condition. Additional exclusion criteria included human immunodeficiency virus infection, prior bariatric surgery, end-stage renal disease, chronic kidney disease stage 4 or 5, and dependence on renal dialysis before the index date. Patients with chronic liver disease were not excluded and remained eligible if they met the study criteria. Liver disease was included as a covariate in propensity score matching. To minimize misclassification of zinc status due to acute illness-related fluctuations, patients with acute kidney failure, critical care services, or pneumonia within one month before the index date were also excluded.

### 2.4 Landmark analysis

A landmark window of 180 days was implemented to limit reverse causation, with the exclusion of any patient who developed sepsis or severe sepsis or who died within this period after the index zinc test. Follow-up for incident sepsis began 180 days after the index date and continued until the earliest outcome occurrence, death, loss to follow-up, or 5 years after the index date.

### 2.5 Propensity score matching

One-to-one propensity score matching was performed using a greedy nearest-neighbor algorithm with a caliper set at 0.1 pooled standard deviations. Propensity scores were estimated using prespecified covariates, including demographics (age, sex, race, and body mass index), comorbidities (e.g., hypertension, dyslipidemia, obesity, neoplasms, vitamin D deficiency, anemia, thyroid disorders, liver disease, ischemic heart disease, iron deficiency anemia, T2DM-related complications, chronic kidney disease, and prior sepsis history), and medications (e.g., corticosteroids, proton pump inhibitors, antilipemic agents, insulin, DPP-4 inhibitors, SGLT2 inhibitors, immunosuppressants, and zinc supplements) (Table 1). Laboratory values were not included in the primary matching model because of incomplete data availability. Covariate balance was evaluated using standardized mean differences (SMDs), with values below 0.10 considered acceptable. Propensity score density plots were inspected before and after matching to confirm adequate cohort overlap. The diagnostic codes used for variables are summarized in Supplementary Table 1.

**Table 1:** Baseline characteristics of patients with low zinc status and controls with normal zinc status before and after propensity score matching.

Variables	Before matching		After matching			
	ZD group (*n* = 10,749)	Control group (*n* = 17,206)	SMD	ZD group (*n* = 9,856)	Control group (*n* = 9,856)	SMD
Patient characteristics						
Age at index (years)	59.4 ± 15.7	57.1 ± 15.2	0.149	58.9 ± 15.8	58.9 ± 15.1	0.003
BMI ≥ 30 (kg/m^2^)	6,356 (59.1)	10,644 (61.9)	0.056	5,907 (59.9)	5,913 (60.0)	0.001
Female	6,573 (61.2)	11,291 (65.6)	0.093	6,171 (62.6)	6,167 (62.6)	0.001
White	6,043 (56.2)	10,123 (58.8)	0.053	5,555 (56.4)	5,579 (56.6)	0.005
Black or African American	2,330 (21.7)	3,653 (21.2)	0.011	2,165 (22.0)	2,148 (21.8)	0.004
Unknown Race	1,395 (13.0)	2,102 (12.2)	0.023	1,276 (13.0)	1,245 (12.6)	0.009
Asian	253 (2.4)	415 (2.4)	0.004	234 (2.4)	236 (2.4)	0.001
Comorbidities and Healthcare Utilization						
Essential (primary) hypertension	7,471 (69.5)	11,727 (68.2)	0.029	6,798 (69.0)	6,849 (69.5)	0.011
Dyslipidemia	6,163 (57.3)	10,794 (62.7)	0.110	5,737 (58.2)	5,762 (58.5)	0.005
Overweight and obesity	5,140 (47.8)	9,268 (53.9)	0.121	4,877 (49.5)	4,879 (49.5)	0.000
Neoplasms	4,044 (37.6)	5,962 (34.7)	0.062	3,627 (36.8)	3,715 (37.7)	0.018
Vitamin D deficiency	3,096 (28.8)	5,549 (32.3)	0.075	2,915 (29.6)	2,949 (29.9)	0.008
Anemias	3,300 (30.7)	3,762 (21.9)	0.202	2,759 (28.0)	2,742 (27.8)	0.004
Disorders of thyroid gland	2,779 (25.9)	4,735 (27.5)	0.038	2,584 (26.2)	2,598 (26.4)	0.003
Diseases of liver	2,886 (26.9)	3,439 (20.0)	0.163	2,430 (24.7)	2,409 (24.4)	0.005
Ischemic heart diseases	2,580 (24.0)	3,394 (19.7)	0.104	2,242 (22.8)	2,248 (22.8)	0.001
Iron deficiency anemia	2,331 (21.7)	2,597 (15.1)	0.171	1935 (19.6)	1921 (19.5)	0.004
Chronic kidney disease (CKD)	1,681 (15.6)	1831 (10.6)	0.148	1,390 (14.1)	1,393 (14.1)	0.001
Nicotine dependence	1,563 (14.5)	2044 (11.9)	0.079	1,329 (13.5)	1,327 (13.5)	0.001
Cerebrovascular diseases	1,512 (14.1)	1879 (10.9)	0.095	1,305 (13.2)	1,313 (13.3)	0.002
Heart failure	1,626 (15.1)	1,600 (9.3)	0.179	1,301 (13.2)	1,297 (13.2)	0.001
T2DM with neurological complications	2088 (19.4)	2,790 (16.2)	0.084	1845 (18.7)	1837 (18.6)	0.002
T2DM with kidney complications	1,313 (12.2)	1,568 (9.1)	0.101	1,107 (11.2)	1,117 (11.3)	0.003
T2DM with ophthalmic complications	795 (7.4)	1,045 (6.1)	0.053	703 (7.1)	706 (7.2)	0.001
T2DM with circulatory complications	719 (6.7)	913 (5.3)	0.058	614 (6.2)	643 (6.5)	0.012
COPD	1,186 (11.0)	1,391 (8.1)	0.100	1,007 (10.2)	1,001 (10.2)	0.002
COVID-19	1,033 (9.6)	1,465 (8.5)	0.038	933 (9.5)	945 (9.6)	0.004
Malnutrition	1,265 (11.8)	1,012 (5.9)	0.209	901 (9.1)	887 (9.0)	0.005
Peripheral vascular diseases	846 (7.9)	983 (5.7)	0.086	703 (7.1)	709 (7.2)	0.002
Alcohol related disorders	875 (8.1)	661 (3.8)	0.182	599 (6.1)	579 (5.9)	0.009
Systemic connective tissue disorders	545 (5.1)	681 (4.0)	0.054	471 (4.8)	480 (4.9)	0.004
Other sepsis	365 (3.4)	373 (2.2)	0.075	291 (3.0)	295 (3.0)	0.002
Unspecified dementia	283 (2.6)	251 (1.5)	0.083	207 (2.1)	218 (2.2)	0.008
Severe sepsis	119 (1.1)	116 (0.7)	0.046	97 (1.0)	96 (1.0)	0.001
Medications						
Corticosteroids for systemic use	6,424 (59.8)	9,710 (56.4)	0.068	5,801 (58.9)	5,857 (59.4)	0.012
Proton pump inhibitors	5,944 (55.3)	8,436 (49.0)	0.126	5,269 (53.5)	5,348 (54.3)	0.016
Antilipemic agents	5,084 (47.3)	8,226 (47.8)	0.010	4,663 (47.3)	4,688 (47.6)	0.005
Insulins and analogues	5,167 (48.1)	6,394 (37.2)	0.222	4,475 (45.4)	4,541 (46.1)	0.013
Biguanides	4,074 (37.9)	7,301 (42.4)	0.093	3,825 (38.8)	3,816 (38.7)	0.002
GLP-1 analogues	1,499 (14.0)	2,873 (16.7)	0.076	1,445 (14.7)	1,474 (15.0)	0.008
Sulfonylureas	1,452 (13.5)	2,266 (13.2)	0.010	1,334 (13.5)	1,327 (13.5)	0.002
DPP-4 inhibitors	1,021 (9.5)	1,651 (9.6)	0.003	945 (9.6)	922 (9.4)	0.008
SGLT2 inhibitors	1,008 (9.4)	1712 (10.0)	0.019	936 (9.5)	948 (9.6)	0.004
Immunosuppressants	887 (8.3)	1,122 (6.5)	0.066	760 (7.7)	775 (7.9)	0.006
Zinc supplementation	699 (6.5)	700 (4.1)	0.109	541 (5.5)	543 (5.5)	0.001

Data are presented as n (%) or mean ± standard deviation. Low zinc status was defined as serum or plasma zinc <0.70 μg/mL, and normal zinc status was defined as 0.70–1.20 μg/mL. Standardized mean differences <0.10 were considered acceptable for covariate balance. BMI, body mass index; CKD, chronic kidney disease; COPD, chronic obstructive pulmonary disease; DPP-4, dipeptidyl peptidase-4; GLP-1, glucagon-like peptide-1; SGLT2, sodium–glucose cotransporter 2; SMD, standardized mean difference; T2DM, type 2 diabetes mellitus; ZD, zinc deficiency.

### 2.6 Primary, key secondary, exploratory, and control outcomes

The primary outcome was incident all-cause sepsis, comprising severe sepsis and other sepsis. Key secondary outcomes included severe sepsis, bacteremia, all-cause mortality, and non-sepsis infection, defined as a composite of pneumonia, urinary tract infection, cellulitis, and abdominal infection.

Individual infection subtypes, including pneumonia, urinary tract infection, and cellulitis, were analyzed as exploratory outcomes to contextualize the non-sepsis infection composite. Follow-up zinc deficiency, defined as a subsequent zinc level <0.70 μg/mL during follow-up, was evaluated as an exposure-consistency outcome to assess whether the baseline zinc classification was reflected in subsequent measurements. Benign skin tumors were used as negative control outcome to assess potential residual confounding or surveillance bias. A list of the diagnostic codes used to ascertain each outcome is presented in the Supplementary Table 2.

### 2.7 Sensitivity, subgroup, and multivariable analyses

Six predefined sensitivity analyses were conducted to evaluate the consistency of primary findings. Model I additionally matched laboratory data, including hemoglobin, C-reactive protein, albumin, hemoglobin A1c, and estimated glomerular filtration rate (eGFR). The inclusion of CRP was intended to account for differences in baseline systemic inflammation between cohorts. Model II excluded patients with a history of sepsis. Model III restricted the analysis to patients with at least one recorded healthcare encounter during follow-up to address potential informative censoring issues. Model IV excluded patients who died during the follow-up period. Model V restricted the study period to 2016–2024. Model VI excluded patients with a history of malnutrition. Subgroup analyses were stratified by sex (male vs. female) and age group (18–65 vs. >65 years), with interaction terms included to test for effect modification. Additionally, multivariable Cox proportional hazards models were applied to the unmatched cohort to identify covariates independently associated with the 5-year risk of incident sepsis.

### 2.8 Exposure-gradient analysis

To examine whether the risk of sepsis scaled with the severity of zinc deficiency, an exposure-gradient analysis was performed. Patients with severe zinc deficiency, defined as zinc levels <0.50 μg/mL, were compared with controls with normal zinc status (0.70–1.20 μg/mL) after propensity score matching to assess whether a lower exposure threshold yielded a larger effect size. The <0.50 μg/mL (50 μg/dL) threshold was selected based on a previous study that identified this concentration as a severe zinc deficiency (28).

### 2.9 Statistical analysis

Time-to-event outcomes were analyzed using Cox proportional hazards models, and the results are reported as hazard ratios (HRs) with 95% confidence intervals (CIs). The primary analysis adopted a cause-specific hazard framework, as the principal objective was to estimate the association between zinc deficiency and sepsis occurrence, rather than the absolute cumulative incidence under competing risks. To assess the potential influence of unmeasured confounding on the primary association, E-values were generated for the point estimate and the lower 95% CI boundary of the association linking low zinc status with incident overall sepsis. To account for the competing event of mortality, a competing risk analysis using the Aalen–Johansen estimator was additionally performed in the full unmatched cohort to estimate the cumulative incidence of sepsis while treating death as a competing event. Fine–Gray subdistribution hazard regression was not available within the TriNetX analytical platform, and individual-level data were not accessible for external model fitting in this study. Adherence to the proportional hazards assumption was examined using Schoenfeld residual analysis. A two-sided *α* of 0.05 was used to define statistical significance for the primary analysis. Given their exploratory purpose, sensitivity analyses, negative/positive control outcomes, and subgroup analyses were reported without correction for multiple testing. All analyses were performed using built-in analytical tools within the TriNetX platform.

## 3 Results

### 3.1 Patient selection and baseline characteristics

**Figure 1:**
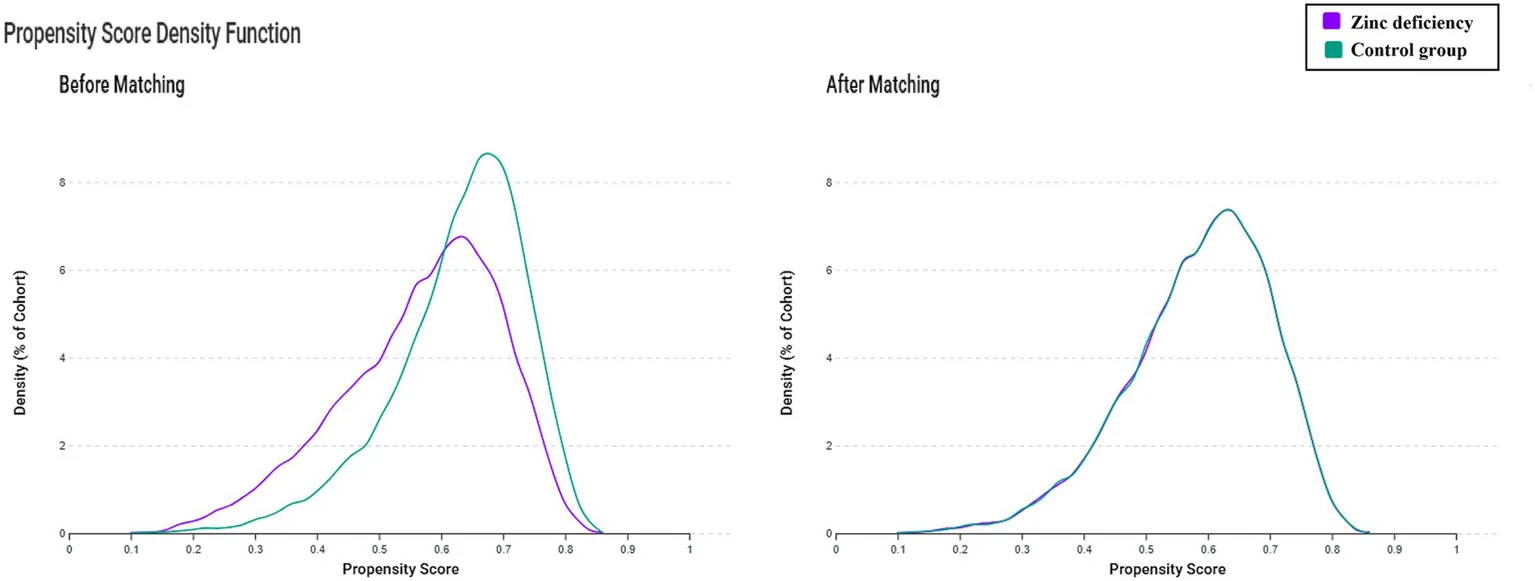
Propensity score density distributions before and after matching. Propensity score density distributions for patients with low zinc status (purple) and controls with normal zinc status (green) are shown before matching (left) and after 1:1 propensity score matching (right). The horizontal axis represents the propensity score, and the vertical axis represents the density as a percentage of each cohort. The nearly complete overlap after matching supports adequate comparability of the measured baseline characteristics between the two groups.

A total of 10,749 patients with low zinc status (<0.70 μg/mL) and 17,206 patients with normal zinc levels (0.70–1.20 μg/mL) were eligible for inclusion. After 1:1 propensity score matching, 9,856 well-balanced pairs were retained for primary analysis. Before matching, several covariates showed notable imbalances, such as anemia (SMD = 0.202), malnutrition (SMD = 0.209), insulin use (SMD = 0.222), and liver disease (SMD = 0.163). After matching, all standardized mean differences were below 0.10, including those for T2DM-related neurological, kidney, ophthalmic, and circulatory complications and glucose-lowering medications, indicating an acceptable covariate balance across demographics, comorbidities, and medications (Table 1). Propensity score density plots confirmed an adequate overlap between the two cohorts (Figure 1).

### 3.2 Primary and key secondary outcomes

In the matched cohort, low zinc status was associated with a higher rate of the primary outcome of overall sepsis (6.86% vs. 5.58%; HR, 1.37; 95% CI, 1.23–1.54; *p* < 0.001) (Table 2). The E-value for the point estimate was 2.08, with a lower confidence interval of 1.76. Among secondary outcomes, low zinc status was also associated with elevated risks of severe sepsis (HR 1.30, *p* = 0.003), bacteremia (HR 1.23, *p* = 0.032), and all-cause mortality (HR 1.56, *p* < 0.001). The association with the non-sepsis infection composite was statistically significant but smaller in magnitude (HR 1.08, *p* = 0.002).

**Table 2:** Association between low zinc status and 5-year risks of incident sepsis.

Outcome	ZD group (*n* = 9,856)	Control group (*n* = 9,856)	HR (95% CI)	*p* value
	Events (%)	Events (%)		
Primary outcome				
Overall sepsis	676 (6.86)	550 (5.58)	1.37 (1.23–1.54)	<0.001
Secondary outcomes				
Severe sepsis	280 (2.84)	239 (2.43)	1.30 (1.10–1.55)	0.003
Bacteremia	234 (2.37)	211 (2.14)	1.23 (1.02–1.48)	0.032
Mortality	916 (9.29)	653 (6.63)	1.56 (1.41–1.73)	<0.001
Non-sepsis infection	3,381 (34.30)	3,463 (35.14)	1.08 (1.03–1.13)	0.002

Data are presented as number of events (%). Non-sepsis infection was defined as a composite of pneumonia, urinary tract infection, cellulitis, and abdominal infection. CI, confidence interval; HR, hazard ratio; ZD, zinc deficiency.

### 3.3 Exploratory infection subtypes, exposure consistency, and negative control outcomes

Among the individual infection subtypes analyzed as exploratory outcomes, pneumonia (HR 1.15, *p* = 0.004), urinary tract infection (HR 1.13, *p* = 0.001), and cellulitis (HR 1.18, *p* = 0.001) showed modest associations with low zinc status, with effect sizes smaller than those observed for sepsis (Table 3). The exposure consistency analysis confirmed that patients initially classified with low zinc status were more likely to have zinc levels below 0.70 μg/mL during follow-up (HR 1.62, *p* < 0.001), supporting the stability of the baseline exposure classification. The negative control outcome, benign skin tumors, showed no significant between-group differences (HR 1.03, *p* = 0.781), suggesting minimal residual surveillance bias.

**Table 3:** Exploratory infection subtypes, exposure-consistency outcome, and negative control outcome.

Outcome	ZD group (*n* = 9,856)	Control group (*n* = 9,856)	HR (95% CI)	*p* value
	Events (%)	Events (%)		
Exploratory infection subtypes				
Pneumonia	939 (9.53)	906 (9.19)	1.15 (1.05–1.25)	0.004
Urinary tract infection	1,659 (16.83)	1,630 (16.54)	1.13 (1.05–1.20)	0.001
Cellulitis	896 (9.09)	844 (8.56)	1.18 (1.07–1.29)	0.001
Exposure consistency outcome				
Follow-up zinc deficiency (<0.70 μg/mL)	1,250 (12.68)	867 (8.80)	1.62 (1.49–1.77)	<0.001
Negative control outcome				
Benign skin tumor	178 (1.81)	192 (1.95)	1.03 (0.84–1.26)	0.781

Data are presented as number of events (%). Follow-up zinc deficiency was defined as a subsequent serum or plasma zinc level <0.70 μg/mL during follow-up. CI, confidence interval; HR, hazard ratio; ZD, zinc deficiency.

### 3.4 Sensitivity analyses

The consistency of the main findings was examined using six sensitivity analyses (Tables 4, 5). The point estimate for overall sepsis remained significant across all models: Model I with additional laboratory matching (HR 1.35, *p* < 0.001), Model II excluding prior sepsis history (HR 1.34, *p* < 0.001), Model III restricting to patients with at least one healthcare encounter (HR 1.44, *p* < 0.001) (Table 4), Model IV excluding patients who died during follow-up (HR 1.37, *p* < 0.001), Model V restricting to 2016–2024 (HR 1.43, *p* < 0.001), and Model VI excluding patients with malnutrition (HR 1.31, *p* < 0.001) (Table 5). The non-sepsis infection composite remained significant, with consistently smaller effect sizes (HR range, 1.06–1.10) across all models. Bacteremia was not consistently significant across sensitivity models, with non-significant results in Model II (*p* = 0.121), Model III (*p* = 0.058), and Model V (*p* = 0.069).

**Table 4:** Sensitivity analyses of the association between low zinc status and risk of incident sepsis: Models I–III.

Outcomes	Model I (*n* = 9,705 for each group)		Model II (*n* = 9,435 for each group)		Model III (*n* = 9,292 for each group)	
	HR (95% CI)	*P* value	HR (95% CI)	*P* value	HR (95% CI)	*P* value
Overall sepsis	1.35 (1.21–1.51)	<0.001	1.34 (1.19–1.51)	<0.001	1.44 (1.28–1.61)	<0.001
Severe sepsis	1.33 (1.12–1.59)	0.001	1.31 (1.09–1.57)	0.004	1.44 (1.21–1.71)	<0.001
Bacteremia	1.21 (1.00–1.45)	0.049	1.17 (0.96–1.42)	0.121	1.19 (0.99–1.43)	0.058
Mortality	1.58 (1.43–1.75)	<0.001	1.54 (1.38–1.71)	<0.001	1.56 (1.40–1.72)	<0.001
Non-sepsis infection	1.09 (1.04–1.14)	0.001	1.09 (1.03–1.14)	0.001	1.10 (1.05–1.15)	<0.001

Model I additionally matched laboratory data, including hemoglobin, albumin, C-reactive protein, hemoglobin A1c, and estimated glomerular filtration rate. Model II excluded patients with any prior history of sepsis. Model III restricted the analysis to patients with at least one recorded healthcare encounter during follow-up. Data are presented as hazard ratios with 95% confidence intervals. CI, confidence interval; HR, hazard ratio.

**Table 5:** Sensitivity analyses of the association between low zinc status and risk of incident sepsis: Models IV–VI.

Outcomes	Model IV (*n* = 9,028 for each group)		Model V (*n* = 9,050 for each group)		Model VI (*n* = 8,596 for each group)	
	HR (95% CI)	*P* value	HR (95% CI)	*P* value	HR (95% CI)	*P* value
Overall sepsis	1.37 (1.19–1.56)	<0.001	1.43 (1.28–1.61)	<0.001	1.31 (1.16–1.48)	<0.001
Severe sepsis	1.43 (1.15–1.79)	0.001	1.45 (1.21–1.73)	<0.001	1.35 (1.11–1.64)	0.002
Bacteremia	1.27 (1.03–1.57)	0.026	1.19 (0.99–1.45)	0.069	1.24 (1.00–1.53)	0.05
Mortality	NA	NA	1.58 (1.42–1.76)	<0.001	1.58 (1.41–1.77)	<0.001
Non-sepsis infection	1.08 (1.03–1.14)	0.003	1.06 (1.01–1.12)	0.014	1.08 (1.03–1.14)	0.004

Model IV excluded patients who died during follow-up. Model V restricted the study period to 2016–2024. Model VI excluded patients with a history of malnutrition. Data are presented as hazard ratios with 95% confidence intervals. CI, confidence interval; HR, hazard ratio; NA, not applicable.

### 3.5 Subgroup analyses

Exploratory subgroup analyses were performed to examine whether the association between low zinc status and incident sepsis differed by sex or age group (Tables 6 and 7). In sex-stratified analyses, the association with overall sepsis was present in both males (HR 1.28, *p* = 0.003) and females (HR 1.44, *p* < 0.001), with no significant interaction (P for interaction = 0.303) (Table 6). In age-stratified analyses, an association with overall sepsis was observed in both the 18–65-year group (HR 1.37, *p* = 0.001) and the older-than-65-year group (HR 1.31, *p* < 0.001), without significant interaction (P for interaction = 0.689) (Table 7). A significant interaction was detected for all-cause mortality (P for interaction = 0.024), with a larger effect estimate in the 18–65-year group (HR 1.99) than in the older-than-65-year group (HR 1.47).

**Table 6:** Sex-stratified subgroup analyses of the association between low zinc status and risk of incident sepsis.

Outcomes	Male (*n* = 3,565 for each group)		Female (*n* = 6,223 for each group)		*P* for interaction
	HR (95% CI)	P value	HR (95% CI)	P value	
Overall sepsis	1.28 (1.09–1.51)	0.003	1.44 (1.23–1.67)	<0.001	0.303
Severe sepsis	1.37 (1.07–1.75)	0.013	1.41 (1.11–1.78)	0.004	0.870
Bacteremia	1.00 (0.76–1.31)	0.979	1.44 (1.12–1.87)	0.005	0.064
Mortality	1.48 (1.28–1.70)	<0.001	1.71 (1.48–1.98)	<0.001	0.167
Non-sepsis infection	1.12 (1.03–1.21)	0.007	1.06 (1.00–1.12)	0.055	0.277

Data are presented as hazard ratios with 95% confidence intervals. *P* for interaction was calculated to assess effect modification by sex. CI, confidence interval; HR, hazard ratio.

**Table 7:** Age-stratified subgroup analyses of the association between low zinc status and risk of incident sepsis.

Outcomes	18–65 years (*n* = 5,203 for each group)		>65 years (n = 5,390 for each group)		*P* for interaction
	HR (95% CI)	*P* value	HR (95% CI)	*P* value	
Overall sepsis	1.37 (1.15–1.63)	0.001	1.31 (1.15–1.49)	<0.001	0.689
Severe sepsis	1.52 (1.15–2.01)	0.003	1.34 (1.10–1.62)	0.004	0.483
Bacteremia	1.28 (0.99–1.65)	0.059	1.20 (0.95–1.50)	0.126	0.715
Mortality	1.99 (1.61–2.46)	<0.001	1.47 (1.32–1.63)	<0.001	0.024
Non-sepsis infection	1.09 (1.01–1.16)	0.018	1.09 (1.03–1.16)	0.003	1.000

Data are presented as hazard ratios with 95% confidence intervals. *P* for interaction was calculated to assess effect modification by age group. CI, confidence interval; HR, hazard ratio.

### 3.6 Multivariable cox regression

In the multivariable Cox proportional hazards model applied to the unmatched cohort (Table 8), low zinc status remained independently associated with incident sepsis (HR 1.47, *p* < 0.001) after adjustment for demographic and clinical covariates. Other variables independently associated with sepsis included male sex (HR 1.48, *p* < 0.001), anemia (HR1.57, *p* < 0.001), malnutrition (HR 1.63, *p* < 0.001), COPD (HR 1.51, *p* < 0.001), chronic kidney disease (HR 1.35, *p* < 0.001), COVID-19 (HR 1.33, *p* = 0.009), liver disease (HR 1.31, *p* < 0.001), and nicotine dependence (HR 1.26, *p* = 0.009).

**Table 8:** Multivariable Cox regression analysis of factors associated with incident sepsis in the unmatched cohort.

Variable	HR (95% CI)	*p*-value
Low zinc status vs. control group	1.47 (1.29–1.67)	<0.001
Male	1.48 (1.30–1.68)	<0.001
Age at index	1.02 (1.01–1.02)	<0.001
White	1.18 (1.03–1.34)	0.014
Dyslipidemia	0.84 (0.73–0.96)	0.012
Overweight and obesity	1.10 (0.96–1.25)	0.182
Neoplasms	1.10 (0.96–1.25)	0.167
Anemias	1.57 (1.37–1.80)	<0.001
Diseases of liver	1.31 (1.14–1.50)	<0.001
Chronic kidney disease (CKD)	1.35 (1.15–1.58)	<0.001
Nicotine dependence	1.26 (1.06–1.50)	0.009
Chronic obstructive pulmonary disease	1.51 (1.26–1.80)	<0.001
Malnutrition	1.63 (1.37–1.94)	<0.001
COVID-19	1.33 (1.07–1.65)	0.009
Alcohol-related disorders	1.10 (0.88–1.38)	0.387
Unspecified dementia	1.24 (0.87–1.77)	0.236

Hazard ratios were estimated using multivariable Cox proportional hazards regression in the unmatched cohort. The variables shown were included in the model as demographic or clinical covariates. CI, confidence interval; HR, hazard ratio.

### 3.7 Exposure-gradient analysis

After propensity score matching, the severe zinc deficiency and normal zinc control groups each included 2,195 patients. Severe zinc deficiency was associated with overall sepsis (HR 1.98; 95% CI, 1.62–2.42) and severe sepsis (HR 2.28; 95% CI, 1.70–3.07). These estimates were larger than those in the primary analysis, suggesting an exposure gradient pattern. The association with non-sepsis infections was weaker (HR 1.21; 95% CI, 1.10–1.34) (Table 9).

**Table 9:** Exposure-gradient analysis of severe zinc deficiency and risks of incident sepsis, secondary outcomes, and non-sepsis infections.

Outcome	Severe ZD group (*n* = 2,195)	Control group (*n* = 2,195)	HR (95% CI)	*p* value
	Events (%)	Events (%)		
Overall sepsis	250 (11.39)	158 (7.20)	1.98 (1.62–2.42)	<0.001
Severe sepsis	125 (5.69)	68 (3.10)	2.28 (1.70–3.07)	<0.001
Bacteremia	114 (5.19)	76 (3.46)	1.83 (1.37–2.45)	<0.001
Mortality	359 (16.36)	210 (9.57)	2.12 (1.78–2.51)	<0.001
Non-sepsis infection	787 (35.85)	803 (36.58)	1.21 (1.10–1.34)	<0.001

Data are presented as number of events (%). Severe zinc deficiency was defined as serum or plasma zinc <0.50 μg/mL, and normal zinc status was defined as 0.70–1.20 μg/mL of serum zinc. Hazard ratios were estimated using Cox proportional hazards models after propensity score matching. Non-sepsis infection was defined as pneumonia, urinary tract infection, cellulitis, or abdominal infection. CI, confidence interval; HR, hazard ratio; ZD, zinc deficiency.

### 3.8 Competing-risk analysis

**Figure 2:**
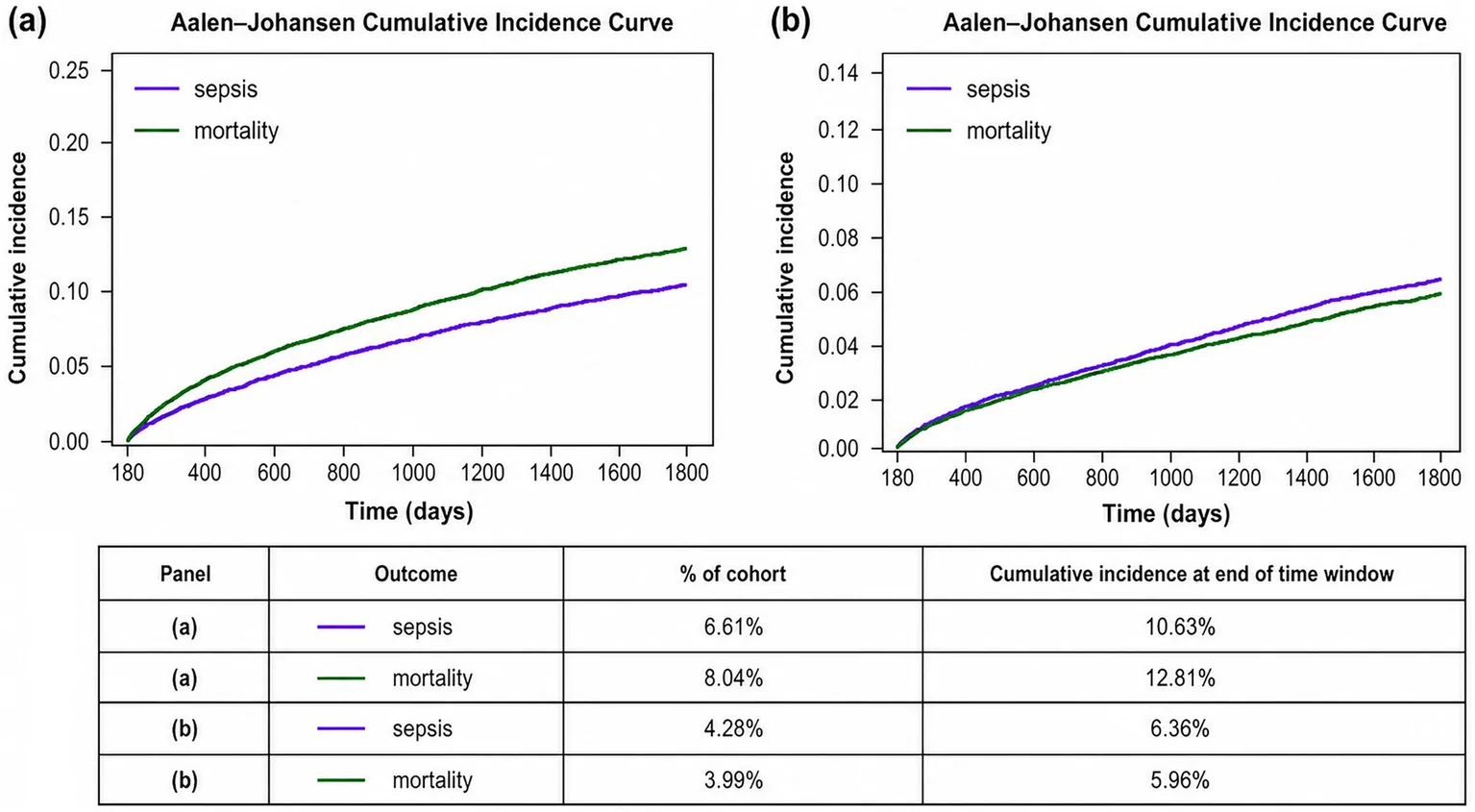
Aalen–Johansen competing-risk analysis of sepsis and mortality. Cumulative incidence curves estimated using the Aalen–Johansen method are shown for sepsis and mortality as competing events in the full unmatched cohort. Panel **(A)** shows patients with low zinc status, and panel **(B)** shows controls with normal zinc status. The table below the curves summarizes the percentage of the cohort and the cumulative incidence at the end of the time window for each outcome. Low zinc status was defined as serum or plasma zinc <0.70 μg/mL, and normal zinc status was defined as 0.70–1.20 μg/mL.

A competing risk analysis using the Aalen–Johansen estimator was performed in the full unmatched cohort to estimate the cumulative incidence of sepsis, while accounting for death as a competing event (Figure 2). At the end of the five-year follow-up, the cumulative incidence of sepsis was higher in the low zinc group (10.63%) than in the normal zinc group (6.36%) (Figure 2). The corresponding cumulative incidence of mortality as a competing event was 12.81% in the low zinc group and 5.96% in the normal-zinc group. Despite the substantially higher competing mortality in the low zinc group, which would be expected to attenuate the cumulative incidence of sepsis, the between-group difference in sepsis incidence persisted throughout the observation period, consistent with the findings of the primary cause-specific hazard analysis.

## 4 Discussion

Using a multicenter retrospective cohort design with propensity score matching and a 180-day landmark, we observed that low zinc status was associated with a higher long-term risk of incident sepsis in patients with T2DM. The findings remained stable across all six prespecified sensitivity analyses, remained significant in the multivariable Cox regression, and showed a similar pattern in the exposure-gradient analysis. Notably, the association appeared stronger for sepsis than for general infections, a pattern that may suggest greater sepsis vulnerability among patients with low zinc status, although this interpretation remains hypothesis-generating.

Several acute care studies have reported that low serum zinc levels during sepsis are associated with greater disease severity, organ dysfunction, inflammatory activation, and higher short-term mortality in critically ill patients (29–31). However, because serum zinc levels may decline during systemic inflammation, these cross-sectional observations cannot determine whether low zinc status precedes sepsis onset or reflects acute-phase zinc redistribution. Moreover, it remains unclear whether pre-existing low zinc status is more strongly associated with sepsis than with non-sepsis infections in patients with T2DM, particularly in longitudinal analyses designed to reduce reverse causation effects.

The central observation of this study was the difference in the magnitude of association between sepsis and non-sepsis infectious outcomes. In the primary analysis, low zinc status was associated with a higher risk of overall sepsis (HR 1.37), whereas the association with non-sepsis infections was weaker (HR 1.08). Individual infection subtypes, including pneumonia, urinary tract infection, and cellulitis, showed modest associations. A similar pattern was observed in the exposure-gradient analysis: severe zinc deficiency was associated with higher risks of overall sepsis (HR 1.98) and severe sepsis (HR 2.28), compared with a smaller association with non-sepsis infections (HR 1.21). These findings suggest that a low zinc status may be more closely related to sepsis vulnerability than to general infection susceptibility. However, sepsis and infection outcomes are not mutually exclusive, and comparisons across outcomes with different baseline risks and ascertainment patterns should be interpreted with caution. Because no formal statistical test was performed to compare effect sizes across outcomes, this pattern should be regarded as hypothesis generating rather than confirmatory.

One possible explanation for this pattern is that zinc deficiency may influence the transition from infection to sepsis by altering pathogen clearance and inflammatory regulation. Sepsis is fundamentally a syndrome of immune dysregulation rather than a direct consequence of pathogen burden (14, 15, 32). Zinc is an important regulator of innate and adaptive immune signaling, including NF-κB-related inflammatory pathways (10–12, 33). Zinc deficiency can promote macrophage inflammatory activation, alter TNF-*α* and IL-6 responses, and impair T cell activation and macrophage phagocytosis (10–12, 33). Although reduced serum zinc levels do not directly establish intracellular zinc depletion, lower zinc availability may weaken ZIP8-mediated zinc inhibition of NF-κB. This process may amplify innate immune activation and proinflammatory cytokine production during infection (34, 35). Animal studies provide complementary mechanistic insights. In experimental diabetic rats, zinc supplementation restored depleted tissue zinc concentrations and modulated the expression of zinc transporters and metallothionein (36). In murine models of polymicrobial sepsis, zinc deficiency exaggerated inflammatory responses, worsened organ injury, and increased mortality, whereas zinc supplementation mitigated these effects (34, 37). These mechanisms may partly explain why a low zinc status was more strongly associated with sepsis than with non-septic infections in this study. This interpretation may be particularly relevant for patients with T2DM, who already exhibit chronic low-grade inflammation and impaired immune regulation (38).

An alternative interpretation is that low serum zinc levels may partly reflect inflammation-driven redistribution rather than depletion of total body zinc. During the acute-phase response, pro-inflammatory cytokines, particularly IL-6, induce hepatic ZIP14 expression in mice. This process increases hepatic zinc uptake and decreases circulating zinc concentrations (9–11, 31). Such redistribution may represent an adaptive response and may make serum zinc a marker of inflammatory burden rather than a systemic zinc deficiency. The exclusion of recent acute illness and the 180-day landmark reduced the likelihood that the index zinc measurement reflected early sepsis. However, chronic or subclinical inflammation in patients with T2DM may have contributed to both low serum zinc levels and subsequent sepsis risk.

The findings on bacteremia provide additional context but should be interpreted with caution. Bacteremia generally requires microbiological confirmation and may, therefore, be less susceptible to diagnostic coding bias than sepsis; however, its ascertainment still depends on whether blood cultures were obtained, which may vary according to illness severity, healthcare utilization, and clinician suspicion. In contrast, sepsis and severe sepsis were ascertained using diagnostic codes, which may have been affected by variations in clinical recognition and documentation. If this misclassification occurred similarly in the low- and normal-zinc groups, it would generally be expected to attenuate the hazard ratios toward the null, potentially underestimating the association. In the primary analysis, low zinc status was associated with a higher risk of bacteremia (HR 1.23), and this association was stronger in the exposure-gradient analysis of severe zinc deficiency (HR 1.83). However, this association was not consistently significant across all sensitivity models, possibly because of the relatively small number of bacteremia events. Taken together, the bacteremia findings are directionally consistent with the sepsis results but remain insufficient to determine whether low zinc status is associated primarily with bloodstream infections, dysregulated host responses, or both. However, this interpretation requires confirmation from studies with larger event counts, blood culture results, and microbiological data.

A competing risk analysis using the Aalen–Johansen estimator was performed as a descriptive analysis to account for death as a competing event. Because all-cause mortality was higher in the low zinc group, differential mortality could have influenced the primary cause-specific hazard analysis if death was treated only as a censoring event. The Aalen–Johansen curves descriptively showed a cumulative incidence pattern consistent with the primary analysis, suggesting that the observed association between low zinc status and sepsis was not driven by differential mortality. However, a formal Fine–Gray subdistribution hazard model (39) could not be performed in the TriNetX analytical environment. Therefore, the Aalen–Johansen findings should be interpreted as supportive and descriptive rather than as substitutes for formal subdistribution hazard analyses. In addition, the 180-day landmark design, while reducing reverse causation, may have introduced survivor bias by excluding patients who died within the landmark period, potentially shifting the analytic cohort toward healthier survivors and under-representing the most vulnerable patients with low zinc status.

Exploratory subgroup analyses suggested that the association between low zinc status and sepsis was present across both sexes and age groups. Formal interaction testing showed no significant effect modification by sex (P for interaction = 0.303) or age group (P for interaction = 0.689) for the primary outcome, indicating that the association with overall sepsis was consistent across subgroups. A significant interaction was detected for all-cause mortality by age group (P for interaction = 0.024), with a larger effect estimate in the 18–65-year group (HR 1.99) than in the older-than-65-year group (HR 1.47). While zinc deficiency is generally considered to have a more pronounced immunological impact in the elderly due to immunosenescence (33, 40), the stronger mortality association in younger patients may reflect the fact that zinc deficiency in younger individuals with T2DM signals a more severe underlying metabolic derangement or poorer overall health status relative to their age-matched peers. However, the mechanisms underlying this age-differential mortality association remain unclear and warrant further investigation.

In the multivariable Cox regression analysis, low zinc status remained independently associated with incident sepsis after adjustment for demographic and clinical covariates (HR, 1.47). Several covariates identified in the model, including anemia, malnutrition, chronic kidney disease, and COPD, may reflect underlying nutritional vulnerability, organ dysfunction, chronic inflammation, or impaired host reserve, all of which could increase susceptibility to infection-related deterioration. These findings support the clinical plausibility of the model but do not eliminate the possibility of residual confounding effects. In patients with T2DM, low zinc status may represent a composite marker of poor glycemic control, renal dysfunction, malnutrition, frailty, chronic inflammatory burden, and impaired host reserve (41–43), rather than an independently modifiable cause of sepsis. The contribution of these factors could not be fully characterized because hemoglobin, HbA1c, eGFR, albumin, and CRP levels were incompletely recorded. Notably, approximately 5.5% of patients in both matched groups received zinc supplements at baseline. As zinc supplementation during follow-up was not fully captured, subsequent initiation or continuation of supplementation may have attenuated the observed association between low zinc status and sepsis risk.

Several limitations should be acknowledged. First, the observational design precludes causal inference, and residual confounding from unmeasured factors, such as dietary zinc intake, frailty, diabetes duration and glycemic control, chronic inflammatory burden, severity of renal or hepatic disease, socioeconomic status, healthcare access, and overall nutritional status, cannot be excluded from the study. As zinc testing is not routinely performed in all patients with T2DM, patients undergoing zinc measurement may have been selected because of suspected nutritional deficiency, chronic illness, or greater healthcare utilization, potentially introducing selection bias (44). Subclinical inflammation at the index date could also have lowered circulating zinc through acute-phase redistribution (45, 46) despite the acute illness exclusions and 180-day landmark, leaving the possibility of reverse causation. Second, laboratory values were not included in the primary matching model due to incomplete data availability, although sensitivity analysis with additional matching for hemoglobin, C-reactive protein, albumin, hemoglobin A1c, and estimated glomerular filtration rate yielded consistent results. However, these laboratory measurements were only available for a subset of patients. Diabetes duration, prealbumin levels, longitudinal glycemic control status, and comprehensive measures of frailty were not consistently captured. Therefore, even after extensive propensity score matching, unmeasured confounding by frailty, nutritional status, metabolic control, and inflammatory burden may account for some of the observed associations. In addition, because individual-level raw laboratory data were unavailable through the TriNetX platform, a direct correlation between serum zinc concentration and CRP could not be evaluated. Third, zinc measurements were generated by local laboratories across participating healthcare organizations, and information regarding the assay methods and preanalytical conditions was unavailable. Therefore, interinstitutional variations in zinc measurements may have introduced exposure misclassification (47, 48). In addition, serum zinc measurements reflect only circulating zinc, which accounts for less than 1% of total body zinc and may not fully capture intracellular zinc status (28, 49). Fourth, sepsis, severe sepsis, and other infection outcomes were identified using ICD diagnostic codes, and some true cases may have been missed or misclassified (50). The washout and landmark strategies improved temporal classification but did not establish the diagnostic accuracy for individual cases. If coding errors occurred similarly in the low- and normal-zinc groups, they would generally be expected to attenuate the hazard ratios toward the null and could, therefore, underestimate the underlying association. However, differential ascertainment related to illness severity or healthcare utilization could have biased the estimates in either direction. Fifth, healthcare organizations within the TriNetX network are primarily drawn from the United States (51, 52), with limited representation of Asian, African, and other populations, where the prevalence of zinc deficiency, dietary patterns, and sepsis epidemiology may differ, limiting the generalizability of these findings.

## 5 Conclusion

These findings suggest that low serum zinc levels may serve as potential markers of sepsis vulnerability in patients with T2DM. The exposure-gradient analysis, in which severe zinc deficiency (<0.50 μg/mL) was associated with a nearly twofold higher risk of sepsis, supports a possible severity-dependent relationship, although this threshold should not be interpreted as a causal cut-off or therapeutic target. Prospective studies and interventional trials are needed to determine whether zinc assessment or correction can improve sepsis risk prediction or reduce the incidence of sepsis in this population.

## Data Availability

The data used in this study were obtained from the TriNetX Global Collaborative Network under institutional access and data-use restrictions. Access to the underlying data requires a direct agreement with TriNetX and approval through the platform’s data access procedures (https://live.trinetx.com). The aggregated, de-identified results generated within TriNetX are available from the corresponding author upon reasonable request.
