# Supplementary material for: Low zinc status and long-term risk of incident sepsis in patients with type 2 diabetes: a multicenter cohort study
Source: Front Nutr. 2026 Aug 21;13:1897312. doi: 10.3389/fnut.2026.1897312 (PMC13543870; doi:10.3389/fnut.2026.1897312)
Supplement: Supplementary file 1 [file Table_1.DOCX]

**Supplemental Table 1. Codes used for variables included in propensity score matching**

| **Variable** | **Code / definition** |
| --- | --- |
| Age | Age at index |
| Sex | Female |
| Race | White, Black or African American, Asian, Unknown race |
| BMI ≥30 kg/m² | BMI-derived structured variable |
| Hypertension | ICD-10-CM: I10 |
| Dyslipidemia | ICD-10-CM: E78 |
| Overweight and obesity | ICD-10-CM: E66 |
| Neoplasms | ICD-10-CM: C00–D49 |
| Vitamin D deficiency | ICD-10-CM: E55 |
| Anemia | ICD-10-CM: D64 |
| Iron deficiency anemia | ICD-10-CM: D50 |
| Thyroid disorders | ICD-10-CM: E00–E07 |
| Liver disease | ICD-10-CM: K70–K77 |
| Ischemic heart disease | ICD-10-CM: I20–I25 |
| Heart failure | ICD-10-CM: I50 |
| Chronic kidney disease | ICD-10-CM: N18 |
| COPD | ICD-10-CM: J44 |
| Cerebrovascular disease | ICD-10-CM: I60–I69 |
| Peripheral vascular disease | ICD-10-CM: I73 |
| Malnutrition | ICD-10-CM: E40–E46 |
| Nicotine dependence | ICD-10-CM: F17 |
| Alcohol-related disorders | ICD-10-CM: F10 |
| Systemic connective tissue disorders | ICD-10-CM: M30–M36 |
| Unspecified dementia | ICD-10-CM: F03 |
| COVID-19 | ICD-10-CM: U07.1 |
| T2DM with kidney complications | ICD-10-CM: E11.2 |
| T2DM with ophthalmic complications | ICD-10-CM: E11.3 |
| T2DM with neurological complications | ICD-10-CM: E11.4 |
| T2DM with circulatory complications | ICD-10-CM: E11.5 |
| Other sepsis | ICD-10-CM: A41 |
| Severe sepsis | ICD-10-CM: R65.2 |
| Insulins and analogues | ATC: A10A |
| Biguanides | ATC: A10BA |
| GLP-1 analogues | ATC: A10BJ |
| SGLT2 inhibitors | ATC: A10BK |
| Sulfonylureas | ATC: A10BB |
| DPP-4 inhibitors | ATC: A10BH |
| Corticosteroids for systemic use | ATC: H02 |
| Proton pump inhibitors | ATC: A02BC |
| Immunosuppressants | ATC: L04 |
| Antilipemic agents | TriNetX medication term: CV350 |
| Zinc supplements | TriNetX medication term: TN450 |

**Supplemental Table 2. Codes used for outcome definitions**

| Outcome category | Outcome | Code / definition |
| --- | --- | --- |
| Primary outcome | Overall sepsis | ICD-10-CM: A41, R65.2 |
| Key secondary outcome | Severe sepsis | ICD-10-CM: R65.2 |
| Key secondary outcome | Bacteremia | ICD-10-CM: R78.81 |
| Key secondary outcome | All-cause mortality | Deceased status; ICD-10-CM: R99 |
| Key secondary outcome | Non-sepsis infection composite | Pneumonia, urinary tract infection, cellulitis, and abdominal infection |
| Exploratory infection subtype | Pneumonia | ICD-10-CM: J12–J18, J69; ICD-9-CM: 483 |
| Exploratory infection subtype | Urinary tract infection | ICD-10-CM: N39.0, N10, N30.0, N30.9 |
| Exploratory infection subtype | Cellulitis | ICD-10-CM: L03 |
| Exploratory infection subtype | Abdominal infection | ICD-10-CM: A00–A09, K35–K38, K57, K63.0, K63.1, K65, K81, K83.0, K75.0 |
| Exposure-consistency outcome | Follow-up zinc deficiency | LOINC: 5763-8; serum or plasma zinc <0.70 μg/mL |
| Negative control outcome | Benign skin tumor | ICD-10-CM: D23 |
